# Cost-Effectiveness and Implementation Strategies for Hypertension Management Using Non-Physician Healthcare Workers in Low- and Middle-Income Countries: A Systematic Review

**DOI:** 10.5334/gh.1533

**Published:** 2026-03-12

**Authors:** Gabriel Lamkur Shedul, Olutobi Adekunle Sanuade, Emmanuel Iroboudu Okpetu, Molly Beestrum, Dike Bevis Ojji, Lisa R. Hirschhorn, Mark D. Huffman, Dustin D. French

**Affiliations:** 1 Department of Medical Social Sciences, Northwestern University Feinberg School of Medicine, Chicago, United States; 2 Cardiovascular Research Unit, University of Abuja, Abuja, Nigeria; 3 Department of Family Medicine, University of Abuja Teaching Hospital, Nigeria; 4 Department of Population Health Sciences, Division of Health System Innovation and Research, Spencer Fox Eccles School of Medicine at the University of Utah, Salt Lake City, UT, United States; 5 Tilman J Fertitta Family College of Medicine, Department of Health System and Population Health Sciences, University of Houston, Houston, United States; 6 Humana Integrated Health Systems Sciences Institute, University of Houston, Houston, United States; 7 Galter Health Sciences Library & Learning Center, Feinberg School of Medicine, Northwestern University, Chicago, Illinois, United States; 8 Havey Institute for Global Health, Northwestern University Feinberg School of Medicine, Chicago, United States; 9 Cardiovascular Division and Global Health Center, Washington University in St. Louis, Saint Louis, United States; 10 School of Public Health Washington University in St. Louis, Saint Louis, United States; 11 The George Institute for Global Health, University of New South Wales, Sydney, Australia; 12 Department of Ophthalmology, Northwestern University Feinberg School of Medicine, Chicago, IL, United States; 13 Department of Veterans Affairs, Center of Innovation for Complex Chronic Healthcare, Hines IL, United States

**Keywords:** hypertension, cost-effectiveness, strategies, task-shifting, low-middle-income countries, non-physician-healthcare workers

## Abstract

**Background:**

This review assessed the cost-effectiveness and implementation strategies of hypertension management by non-physician healthcare workers (NPHCWs) in low- and middle-income countries (LMICs).

**Methods:**

A systematic search (inception–May 2024) included adults ≥18 years managed by NPHCWs LMICs, following Preferred Reporting Items for Systematic Reviews and Meta-analyses (PRISMA) guidelines. Economic evaluations were assessed using Drummond’s checklist and ROBINS-I.

**Results:**

Seven studies (2002–2022) conducted across eight countries enrolled 96–10,000 participants and included randomized, modeling, observational, and quasi-experimental designs. NPHCWs included pharmacists, community and village health workers, and nurses. Patients’ mean age ranged 58–71 years, with 57–82% female. Outcomes assessed included cost per mmHg reduction ($INT 2.25 systolic, $INT 2.03 diastolic), per controlled patient ($INT 1.48), annual cost ($INT 0.22–232.31), cost per disability-adjusted life year (DALY) averted ($INT 411.39–4709.96), and per quality-adjusted life year (QALY) gained ($INT 1.04–13.30). Incremental cost-effectiveness ratio (ICERs) varied ($INT 0.41–14,373.97). Strategies included NPHCWs training and community engagement/counseling.

**Conclusion:**

Hypertension management by NPHCWs appears cost-effective in LMICs, though more studies are needed for generalizability.

## Introduction

Hypertension is a common and rapidly increasing non-communicable disease (NCD), affecting more than 1 billion adults in low and middle-income countries (LMICs) (1,2). Annually, it accounts for 10.8 million preventable deaths and 234 million years of life lost or lived with a disability (1). Despite its high burden, only 46% of individuals with hypertension are aware of their condition, 42% are diagnosed and treated, and only 15.4% achieve control (3,4). These gaps in the hypertension cascade of care can be attributed to factors such as limited education and access to quality healthcare, limited funding, and health workforce shortages in LMICs, posing substantial challenges to hypertension management in these countries (5,6).

Recognizing the severity of this problem and the socioeconomic burden of NCDs, the United Nations has set a target to achieve a one-third reduction in premature mortality from NCDs, including hypertension, from 2010 to 2030 (3,7). However, translating this target into effective implementation in resource-poor settings largely depends on the cost of implementation (8). Recognizing that understanding costs is critical for government to support and fund the control of NCDs such as hypertension in LMICs, the World Health Organization (WHO), and the United Nation (UN) encourages countries to conduct economic evaluations of the potential benefits of increasing their NCD responses (9). Although cost-effectiveness is an integral part of scaling up effective hypertension care and is critical to achieving universal health coverage, data from LMICs remain limited (10,11). Even where cost is not a primary barrier, there remains a significant disparity between expert recommendations and actual blood pressure (BP) control in practice (12,13). Bridging this gap requires effective implementation strategies that can ensure optimal blood pressure management and control. Scaling up these strategies could minimize hypertension-related morbidity and mortality in LMICs (13,14).

Managing hypertension in LMICs with physicians is generally cost-effective (8). However, due to a shortage of physicians in these regions, task shifting (i.e., a strategy that assign some responsibilities to lower-skilled healthcare workers where needed for maximal use of human resources for expanded healthcare while training for long term and retention is done) and task sharing (i.e., a collaborative distribution of tasks where responsibilities are shared among team members or across cadres of healthcare workers who have the potential to offer suitable healthcare services) to non-physician healthcare workers (NPHCWs) is increasingly implemented into routine practice (15,16,17,18). NPHCWs often undergo short-term training with basic qualifications to handle tasks such as hypertension evaluation and management under the supervision of a physician (19,20,21). It is crucial to examine the strategies used to support task shifting or sharing to NPHCWs, and the strategies in implementing task shifting and task sharing to improve blood pressure control, patient outcomes, and strengthen health systems in LMICs. There is limited evidence on the cost-effectiveness and the strategies used for managing hypertension by NPHCWs in LMICs, which provides the rationale for this study. This study aims to contribute to the body of knowledge by systematically reviewing the evidence on costs and cost-effectiveness and by describing the strategies used to support task shifting and its implementation in blood pressure control by NPHCWs in LMICs to inform policy formulation that will improve access and quality of hypertension care.

## Methods

A study protocol was developed and registered in PROSPERO (CRD42024544755) before starting the review. The Preferred Reporting Items for Systematic Reviews and Meta-analyses (PRISMA) and reporting guidelines for economic evaluations of biomedical literature were followed to conduct this review (22,23). To guide the search process, we developed inclusion and exclusion criteria following PICOTS (Population, Intervention, Comparator, Outcomes, Time course, and Study design), detailed in **Supplemental Table 1**. The inclusion criteria for this review were adults ≥ 18 years managed for hypertension by NPHCWs (e.g., pharmacists, community health workers, nurses) in LMICs. The interventions were hypertension management, both pharmacological (e.g., use of BP-lowering medications), and non-pharmacological (e.g., lifestyle modifications such as weight reduction, regular physical activity, smoking cessation, and maintaining a healthy diet). The comparator was usual care for the given context. The outcomes include: (1) costs per 1 mm Hg reduction in BP (systolic BP, diastolic BP, or both); (2) cost of controlling hypertension (<140/90 mmHg) per patient; (3) the costs of managing patients with hypertension by NPHCWs; (4) disease impact on quality of life (cost per disability averted life year); and (5) cost per quality of life gained. We also extracted data on the incremental cost-effectiveness ratio and standardized costs based on the per capita gross domestic product of the country where the study was conducted (see **Supplemental Table 2** for definition of terms) as well as strategies used to support task shifting and those implemented in blood pressure. Studies conducted before May 2024 that met the inclusion criteria were included in the review process. Additional criteria included publications in English that utilized primary or simulation data to assess cost-effectiveness of hypertension management by NPHCWs in adults aged 18 years and above in LMICs. We excluded abstracts, opinion letters, editorials, and studies involving surgical interventions. Successful evidence-based interventions can produce desired outcomes when the right implementation strategies are correctly chosen and applied in the right context (13). These strategies can be applied at system, organizational, or individual levels (24). Using Proctor’s conceptual framework of implementation research, the outcomes can be implementation outcomes (e.g., costs) (25), service outcomes (e.g., effectiveness), or client outcomes (e.g., function and symptomatology) (26).

Published articles from inception to May 2024 were searched by a medical librarian (MB) from five bibliometric databases: Ovid Medline, Embase, Cochrane, CINAHL, and Scopus. The Medical Subject Headings (MeSH) related to cost-effectiveness and strategies of hypertension management using NPHCWs in LMICs were used by MB as a search strategy to pull related articles. A complete record of the MESH searched terms used can be found in **Supplemental Table 3**. A complete gray literature search was not done since a preliminary search did not yield any significant findings. Two independent reviewers (LGS and OAS) did the screening with 95% agreement before unblinding to resolve the 5% of titles and abstracts where there was disagreement through discussion. The full texts were retrieved and screened independently by two reviewers (LGS and OAS). Relevant data were extracted from screened full text into a data collection form. The data collection tools adapted included cost-effectiveness questionnaire for hypertension management and Actor-Action-Target-Dose framework for implementation strategies.

An intervention was considered cost-effective when the costs were less than three times the annual per capita gross domestic product (GDP) of the respective country, and very cost-effective when the costs of the intervention is less than or equal to the annual per capita GDP for that country where the study was done (8,27,28). For the multi-country study, costing was done separately for each country before conversion to international dollars ($INT). The cost-effectiveness was assessed by considering the cost of intervention in each country against their respective GDP. For uniformity and ease of comparison, costs were adjusted for inflation to 2022 of their individual country’s currencies using global rates inflation calculator (29). The adjusted costs were then converted to $INT using the World Bank Purchasing Power Parity conversion factor (30). When a study did not report the country’s GDP, a corresponding nominal GDP per capita for the year 2022 accessed online from Worldometer was used for the above assessment (31). Cost consideration included medications, lifestyle modifications, equipment, relevant investigations, labor, transportation, healthcare workers’ training, and time spent offering healthcare, health education, and healthcare workers’ stipends. Proctor’s conceptual framework of implementation research was used in this review because it gives a better way to conceptualize and assess implementation (26). The strategies used were grouped into those that support task shifting to NPHCWs and those implemented by the NPHCWs to support patients in controlling their blood pressure. Other data extracted included country group by World Bank classifications, country of research, authors, year of publications, age and sex of participants, study period, study design, sample size, care provider, population, intervention, comparator, study period, sources of funding and declaration of conflicting interest. Missing data or any unclear information was excluded from the analysis. A narrative synthesis was conducted based on the heterogeneity of findings.

Quality of each included study report was independently assessed using the 10-item Drummond guidelines for economic evaluation of healthcare programs by two authors (LGS and OAS), and discrepancies were resolved by discussion. Assessments evaluated clarity on the study question, study description/intervention, study design, identification, measurement, and valuation of costs and consequences. Additional assessments included whether discounting was performed, whether incremental cost-effectiveness ratio (ICER) analyses were conducted, whether results were reported with measures of uncertainty and sensitivity analyses, and how the findings were interpreted.. A “yes” answer to each domain was scored 1, and a “no” answer was scored 0. A final score of 1 to 3 was assessed as low quality, 4 to 7 was moderate quality, and 7 to 10 was assessed as high quality (23,32,33).

Risk of bias assessment was done using the ROBINS-I tool, which is a 7-item assessment tool to estimate the effectiveness or safety of an intervention from non-randomized studies (34). The tool assesses bias due to confounders, selection of study participants, classification of interventions, deviation from interventions, missing data, measurement of outcomes, and selection of reports. The risk of bias assessment was categorized as low, moderate, serious, and critical for each domain. A study with all the domains having low risk of bias was judged as low risk, while a study that combines low and moderate risk of bias was assessed to be low or moderate risk of bias. However, if a study had at least one domain rated as having a serious or critical risk of bias, the overall risk of bias for that study was judged as serious or critical, respectively (34).

**Patient and public involvement**: There was no patient or public involvement in the process of this systematic review.

## Results

### Study characteristics

**Figure 1:**
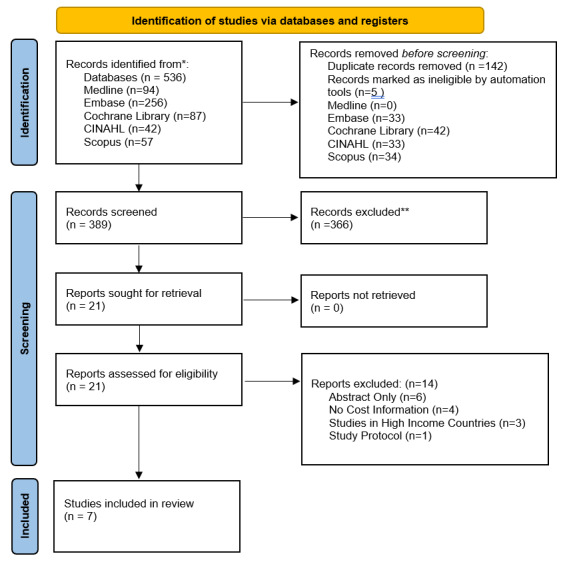
PRISMA 2020 flow diagram for new systematic reviews which include searches of databases and registers only.

The PRISMA flowchart is shown in Figure 1. From the search of five databases, a total of 536 studies were identified. After de-duplication and an initial screening of titles and abstracts, 21 full texts were reviewed. Following the full-text review, six studies were excluded due to having only abstracts available, four studies did not have information on costs, and three studies were conducted in high-income countries. Additionally, one study was identified as study protocol. Ultimately, only seven studies from eight countries with full text met the inclusion criteria in the analysis. The list of included studies is shown in **Supplemental Table 4**. Four of the included studies were from upper-middle-income countries (two studies from Indonesia, Mexico, and South Africa) (35,36,37,38), two studies were from LMICs (Ghana and a multinational study involving Bangladesh, Pakistan, and Sri Lanka) (39,40), and one from low-income country (Uganda) (Table 1) (41). Only two studies included medication costs (39,41) while the remaining costs were on training, health education, screening, equipment, laboratory tests, stipends to healthcare providers, and healthcare providers’ time (35,36,37,38,40). Study designs were randomized controlled trials (*n* = 2) (35,39), modeling (*n* = 2) (36,40), observational (*n* = 2) (37,41), and quasi-experimental studies (*n* = 1) (38). NPHVWs included pharmacists (37,38,40) (*n* = 3), community health workers (36,39) (*n* = 2), nurses (35) (*n* = 1), and village health workers (38) (*n* = 1). The mean age for patients ranged from 58 ± 12.1 to 70.8 ± 8 years, with the proportion of female patients ranging from 57% to 82%.

**Table 1:** Study characteristics of included studies.


AUTHORS	FINKELSTEIN ET AL. (39)	GARCIA-PENA ET AL. (35)	GAZIANO ET AL. (36)	POZO-MARTIN ET AL. (40)	RIWU ET AL. (37)	STEPHENS ET AL. (41)	YUSRANSYAH ET AL. (38)

**Country Group**	Lower-Middle Income Countries	Upper-Middle Income Countries	Upper-Middle Income Country	Lower-Middle Income Country	Upper-Middle Income Country	Lower-Income Country	Upper-Middle Income Country

**Country(ies)**	Bangladesh, Pakistan, Sri Lanka	Mexico	South Africa	Ghana	Indonesia	Uganda	Indonesia

**Publication year**	2021	2002	2014	2021	2019	2021	2022

**Study period (Duration)**	April 2016–March 2019 (3 years)	Jan 1998–Jun 1999 (1 year)	1 year modelling	10 years modelling	Dec 2017–Feb 2018 (3 months)	2011–2021 (10 years)	Jun–Aug 2019 (3 months)

**Study design**	RCT	RCT	Modelling	Modelling	Observational	Descriptive observational study	Quasi-experimental study

**Sample size**	2645	718	525	10000	100	413	96

**Care provider**	Community Health Workers	Nurses	Community Health Workers	Pharmacists & Nurses	Pharmacists	Village Health Workers	Pharmacists

**Female /Male**	NI	459/259	NI	NI	57/43	NI	77/19

**Population**	≥ 40 years	≥ 60 years	25–74 years	18–79 yeas with hypertension	Hypertensive patients	30–69 years with hypertension	Adults with hypertension

**Intervention**	Multicompetent interventions (Home health education, training of healthcare providers on diagnosis, treatment, follow-up & referral of hypertension, hypertension triaging, financial compensation for additional services)	Home visits for health & lifestyle advice to participants with hypertension	Home visits & hypertension education	Health Education, screening, diagnosis & management of hypertension among community members & referral of severe cases. Information, communication technology messages on healthy lifestyles, treatment adherence & medication refills	Counselling of patients by pharmacists	Screening, diagnosis & treatment of hypertension including counselling on healthy lifestyles	Counselling by pharmacists

**Comparator**	Usual care which is home visit for maternal & childcare	Controlled group received mailed pamphlet about hypertension	Usual or existing care	Standard care (hypertension management services by Ghana Health Services at the time of the study)	No counselling by pharmacists	None	No counselling by pharmacists

**Outcomes**	The program was cost-effective in all the 3 countries with a cost-effectiveness acceptability curve predicted 79.3% cost-effective in Bangladesh, 85.2% in Pakistan, & 99.8% in Sri Lanka	The reduction in BP was cost-effective	Cost-effective at reducing CVD & number of clinic visits	Not cost-effective because of high overhead & high patient cost.	Cost-effective in BP control & improved quality of life.	Cost-effective with BP control	Cost-effective at optimizing hypertension treatment at the PHCs.

**Sources of funding**	UK department of Health & Social Care, UK department of International Development, the Global Challenge Research Fund, UK Medical Research Council, Wellcome Trust	National Council of Science & Technology Mexico, Mexican Institute of Technology	NI	Novartis Foundation	Research Development of Ministry of Research, Technology and Higher Education Republic of Indonesia	None	Ministry of Research, Technology and Higher Education Republic of Indonesia

**Conflict of interest declaration**	None	NI	None	Some co-authors received grants from Novartis Foundation, while others received funds from Novartis.	None	None	None


NOTE: RCT- Randomized Controlled Trials, NI – No information, CVD – Cardiovascular Diseases, PHCs – Primary Healthcare Centers. BP – Blood Pressure.

### Cost and cost-effectiveness

The individual studies reported various cost components as shown in Table 2. One study reported a cost of $INT2.25 per 1 mmHg reduction in SBP (35), and $INT2.03 per 1 mmHg reduction in DBP (35). Another study reported $INT1.48 as the costs of controlling hypertension (BP < 140/90 mmHg) per person (37). Five studies reported annual costs of treating one person with hypertension ranging from $INT 0.22 to $INT 232.31. Three studies reported costs per averted disability-adjusted life year (DALY) from $INT411.39 to $INT4709.96. A study by Riwu et al. reported costs per quality-adjusted life year (QALY) gained from only payers’ perspective ($INT1.04) while another by Yusransyah et al. reported it from both payers’ ($INT172.88) and patients’ perspectives ($INT13.30) (37,38). Five studies were deemed very cost-effective as their DALY, QALY, and ICER values were below their respective countries’ GDP (35,36,37,38,39,41). Reported absolute cost-effectiveness included: $INT 2.25 per 1 mmHg reduction in SBP, $INT 430.48 per DALY averted, $INT 869.77 per DALY averted, $INT 0.22 per person per year, and $INT73.01 per QALY gained. Their corresponding relative cost-effectiveness values expressed as a percentage of national GDP per capita were 0.01%, 2.71%, 15.22%, 0.02%, and 1.200%, respectively. The multinational study reported absolute cost-effectiveness values of $INT 3959.60 for Bangladesh, $INT 2620.49 for Pakistan, and $INT 4709.96 for Sri Lanka. The relative cost-effectiveness was 128%, 142%, and 120%, respectively. In contrast, the modeling study by Pozo-Martins et al. with the intervention of training, screening, diagnosis, treatment, and counselling in Ghana was not cost-effective. The absolute and relative cost-effectiveness were $INT 14373.97 or 504% of Ghana’s GDP per capita, which is greater than the country’s willingness to pay and three times the per capita GDP (40). Three studies were reported from the payers’ perspective (35,37,39), one from the societal perspective (40), one from both payer and patient perspectives (38). Two studies did not document the perspective (36,41). Only the modeled studies reported ICER (36,40). Generally, the interventions were found to be cost-effective except for Pozo-Martins et al. (2021) which was a modeling study that found that the intervention (training of community pharmacists and nurse-led hypertension management, community education, BP screening and monitoring, cloud based health record system, adherence support and referral system) was not cost-effective due to high overhead and patient cost (40).

**Table 2:** Costs of hypertension treatment.


AUTHORS	FINKELSTEIN ET AL.	GARCIA-PENA ET AL.	GAZIANO ET AL.	POZO-MARTIN ET AL.	RIWU ET AL.	STEPHENS ET AL.	YUSRANSYAH ET AL.

**Country(ies)**	Bangladesh, Pakistan, Sri Lanka	Mexico	South Africa	Ghana	Indonesia	Uganda	Indonesia

**Cost items**	Labor, rentals, materials & supplies, contracted services	Training nurses, equipment, office space, supplies, & time spent travelling & attending to patient	Salaries of community health workers & program coordinators, training (per diem for trainers, trainees, room rentals, chairs, desks, laptop computers, projector, projector screen, notebook, pencil), home visits & follow-up (cell phones & minutes, BP apparatus, recording sheets, educational pamphlets)	Training, screening, administrative support, equipment, investigations, treatments, follow-up, electronic health records	Costs of managing hypertension including counselling	Hypertension screening, diagnosis, treatments & stipends to village health workers	Antihypertensive medication, capitations rate by social health insurance administrative body

**Perspective(s)**	Payer (Health Ministry)	Payer (Health service) & patient	Healthcare Worker	Societal	Payer (Health insurance organization)	NI	Payer & patient

**Year of costing in USD**	2019	1998	2012	2017	2017	NI	NI

**Cost of 1 mmHg reduction in SBP**	NI	$1.14 ($INT2.25)	NI	NI	NI	NI	NI

**Cost of 1 mmHg reduction in DBP**	NI	$1.03 ($INT 2.03)	NI	NI	NI	NI	NI

**Cost of controlling Hypertension per person**	NI	NI	NI	NI	IDR 6387 ($INT 1.48)	NI	NI

**Annual Cost of treating 1 person**	Bangladesh $10.65 ($INT 12.70), Pakistan $10.25, ($INT 11.54), Sri Lanka $6.42 ($INT 6.93)	$ 7.56 ($INT 14.50)	$8 ($INT 10.28)	$197.20 ($INT 232.31) Current CommHIP, $152.10 ($INT 179.25) GHS-LCS CommHIP	No information	$0.20 ($INT 0.22)	IDR 17,923/patient/counselling for government salary (Annual costs IDR 215,076) ($INT 46.92)

**Cost per averted DALY**	$3430 ($INT 3959.60) in Bangladesh, $2270 ($INT 2620.49) in Pakistan, & $4080 ($INT 4709.96) in Sri Lanka	NI	$320 ($INT 411.39)	$645 ($INT 770.08)	NI	NI	NI

**Cost per gained QALY**	NI	NI	NI	NI	4490 IDR ($INT 1.04)	NI	IDR 850185 ($INT 172.88) (payers’ perspective), IDR 65394.15 ($INT 13.30) (patient’s perspective)

**ICER**	NI	NI	$335 ($INT 430.48)	$12189 ($INT 14373.97) Current CommHIP, $6530 ($INT 7700.55) GHS-LCS CommHIP	IDR 2296 ($INT 0.53)	NI	IDR 2000–28307 ($INT 0.41 –5.76)

**GDP**	Bangladesh $2,688*, Pakistan $1,597* Sri Lanka $3,408*	$11,091*	$12,855.82	$2,388	$4,788*	$964*	$4,788*

**Absolute Cost-effectiveness** ^††^	Bangladesh $3430 ($INT 3959.60)/ DALY averted Pakistan {$2270 ($INT 2620.49)/ DALY averted} Sri Lanka {$4080 ($INT 4709.96)/ DALY averted}	$1.14/ 1mmHg SBP reductions	$335 ($INT430.48)/ DALY averted	$12189 ($INT14373.97)/ DALY averted	$728.5 / DALY averted	$0.20/ person/ year	$57.26/ QALY gained

**Relative Cost-effectivenessǂǂ(% of GDP per capita)**	Bangladesh 128% (cost-effective) Pakistan 142% (cost-effective) Sri Lanka 120% (cost-effective)	0.01% (very cost-effective)	2.71% (very cost-effective)	504.00% (not cost-effective)	15.22% (very cost-effective)	0.02% (very cost-effective)	1.20% (very cost-effective)


NOTE: Costs in brackets are converted to 2022 $INT using the World Bank Group Purchasing PowerSS Parity conversion factor (30). NI: No Information given. GDP: gross domestic product in 2022. ICER: incremental cost-effectiveness ratio. $INT: international Dollar. QALY: quality-adjusted life year. DALY: disability-adjusted life year. mmHg: millimeters mercury. SBP: systolic blood pressure. DBP: diastolic blood pressure. IDR: Indonesian Rupiah. $* Corresponding countries nominal GDP per capita for 2022 from online Worldometers (31). USD: United States Dollar. GHS-LCS CommHIP: Ghana Health Service-Licensed Chemical Sellers Community-based Hypertension Improvement Project. ^††^Absolute cost-effectiveness is the total cost in US dollars required to gain one additional unit of health outcomes, such as QALY, DALY averted, 1 mmHg reduction in SBP or DBP and ICER. ǂǂRelative cost-effectiveness is the ratio of the ICER or DALY averted or QALY gained or 1 mmHg reduction in SBP or DBP to the individual country’s GDP per capita.

### Implementation strategies

Several strategies were used across different studies with some studies using multiple strategies as shown in Table 3. Studies that used strategies to support task shifting included four studies that used training and education, two studies used financial strategies (39,41), one study implemented a change in infrastructure (40), and another one deployed a cloud-based electronic medical record. Strategies used by the NPHCWs to support patients care or improve BP control include three studies that used community engagement and counselling on medication adherence and lifestyle modifications. Another strategy used by NPHCWs involved Information and Communication Technology (ICT), including Short Message Services (SMS), to promote healthy living and provide medication refill reminders for hypertension treatment. Community health workers, nurses, pharmacists, and village health workers were the key actors who implemented these strategies. All studies reported that more than one implementation outcome was affected by the strategies. Costs and cost-effectiveness were the most common implementation and service outcomes reported.

**Table 3:** Specification of Strategies used in task shifting to non-physician healthcare workers and Implementation of Task Shifting by non-physician healthcare workers (65).


AUTHORS	FINKELSTEIN ET AL.	GARCIA-PENA ET AL.	GAZIANO ET AL.	POZO-MARTIN ET AL.	RIWU ET AL.	STEPHENS ET AL.	YUSRANSYAH ET AL.

**Name of strategy(ies)**	Train, education of stakeholders & utilization of financial strategies	Train & educate stakeholders, engage consumer	Train & educate stakeholders	Train & educate stakeholders, engage consumers & change infrastructures	Engagement of patients through counselling.	Train & educate stakeholders, engage consumes, use financial strategy	Engage consumers

**Definition/ description of strategy(ies) used in task shifting to non-physician healthcare workers**	The training involve education of healthcare workers in the diagnosis and management of hypertension. The financial model used here was to secure additional funding to compensate for up to 20% of healthcare workers’ salaries.	Training nurses about ageing, diagnosis & management of hypertension, how to conduct personal interviews, behavioral change models & ethics of home visits.	Train community health workers on using semi-automated BP measuring apparatus & also on etiology & prevention of hypertension & cardiovascular diseases. Documentation & monitoring of patient’s BP, treatment adherence & health education on healthy lifestyles with referrals where necessary.	Provision of cloud-based health record system linked to Short Message Service (SMS)/voice messaging for treatment adherence, reminder & health messaging.		Training village health workers on diagnosis & treatment of hypertension including healthy lifestyle. Village health workers are paid stipend as incentive per patient recruited or follow-up.	

**Definition/ description of strategy(ies) used by non-physician healthcare workers to improve patients’ outcomes**	Education on hypertension & risk factors was done to patients by healthcare workers during home visits	During visits, nurses & patients reviewed health records from baseline changes & discussed the possible lifestyle changes. Two to four weekly subsequent visits were discussed & approved at the patient’s’ convenience.		Community education on cardiovascular risk factors & healthy lifestyles, BP screening & monitoring by community pharmacists & cardiovascular diseases nurses, diagnosis, treatment, counselling, follow-up & referrals for severe cases. Information, Communication and Technology (ICT) messages on healthy lifestyles, treatment adherence support & reminders for hypertensive drug refills.	Counselling of patients with hypertension on medications by pharmacist during clinic visits	Village health workers go from house to house twice a year to engage & screen adults 25 years & above for hypertension & monthly follow-up and referral of severe cases to nurses supervising them.	Pharmacists engage patients by counselling to enhance treatment adherence resulting in improvement of blood pressure & quality of life.

**Actor: Identify who enacts the strategy**	Healthcare Workers	Nurses	Community Health Workers	Licensed Chemical Sellers and Cardiovascular Diseases Nurses	Pharmacists	Village health workers	Pharmacists

**Action: specify the steps, actions or process to be taken**.	Health education, training & management of hypertension	Home visit, blood pressure measurements and healthy lifestyle counselling	Education of patients on risk of hypertension & benefits of lifestyle changes & medication adherence	Community-based education on cardiovascular diseases risk factors & healthy lifestyles, screening, diagnosis, treatment, counselling & referral of severe cases. Information, communication & technology messages for healthy lifestyles, treatment adherence, & medication refill reminders, cloud-based health record system	Counselling on antihypertensive medications	Training, screening, diagnosis, counselling on healthy lifestyle, treatment & follow-up of enrollees with hypertension	Counselling

**Action Target: Who or where the strategy is directed or targeted to impact**	Patients	Patients	Patients	Community members and patients	Patients	Patients	Patients

**Temporality: When/sequence of use**	Pre-implementation & during implementation (training providers, follow-up, treatment, sustained over 2 years	Exploration/ adoption phase to implementation phase	Exploration to implementation phase	Blended community-facility-based model that spans adoption, implementation & sustainment phase	Implementation phase	Exploration/adoption to implementation with community BP care & follow-up	National program sustainment phase

**Dose: Frequency/intensity/duration of strategy used**	3-monthly home education & BP screening. Each session lasting 30–90 mins	Intervention is 2–4 weekly & lasted for 6 months	2 times a year	No information	Once during clinic visits lasted 15–30 mins	Twice yearly screening & monthly treatments & follow-up	Monthly education/clinic visits & 3 monthly laboratory tests

**Implementation outcomes likely to be affected**	Reach, adoption, fidelity, costs & effectiveness	Reach, adoption, fidelity, costs & effectiveness	Reach, adoption, fidelity, costs & effectiveness	Reach, adoption, fidelity, costs & effectiveness	Reach, adoption, fidelity, costs & effectiveness	Reach, fidelity, adoption, costs & effectiveness	Reach, adoption, fidelity, costs, & effectiveness


BP: Blood pressure. SMS: Short Message Service. ICT: Information Communication Technology.

### Quality assessment

Based on the 10-item checklists of Drummond’s guidelines for quality of economic evaluation, most of the included studies had scores of ≥ 9, except for the two observational studies that had scores of 7 and 3, respectively (Table 4).

**Table 4:** Assessments of quality of studies on economic evaluation using Drummond checklist.


QUESTION	FINKELSTEIN ET AL.	GARCIA-PENA ET AL.	GAZIANO ET AL.	POZO-MARTIN ET AL.	RIWU ET AL.	STEPHENS ET AL.	YUSRANSYAH ET AL.

**1. Was a well-defined question posed in answerable form?**	1	1	1	1	1	1	1

**2. Was a comprehensive description of the competing alternative given? (i.e. Can you tell who? did what? to whom? where? & how often?)**	1	1	1	1	1	1	1

**3. Was there evidence that the program’s effectiveness had been established?**	1	1	1	1	1	1	1

**4. Were all the important & relevant costs & consequences for each alternative identified?**	1	1	1	1	1	0	1

**5. Were costs & consequences measured accurately in appropriate physician units? (e.g. hours of nursing time, number of physician visits, lost workdays, gain life years)**	1	1	1	1	1	0	1

**6. Were costs & consequences valued credibly?**	1	1	1	1	1	0	1

**7. Were costs & consequences adjusted for differential timing?**	1	0	0	1	0	0	0

**8. Was an incremental analysis of costs & consequences of alternatives performed?**	1	1	1	1	1	0	1

**9. Was uncertainty in the estimates of costs & consequences adequately characterized?**	1	1	1	1	0	0	1

**10. Did the presentation & discussion of study results include all issues of concern to users?**	1	1	1	1	0	0	1

**Overall score**	**10**	**9**	**9**	**10**	**7**	**3**	**9**


### Risks of bias assessment

ROBINS-I Assessment tools showed that two studies had a low risk of bias, three had a moderate risk of bias, and another two had a serious risk of bias (Table 5).

**Table 5:** Assessment of risk of bias of included studies using ROBINS-I tool.


QUESTIONS	FINKELSTEIN ET AL.	GARCIA-PENA ET AL.	GAZIANO ET AL.	POZO-MARTIN ET AL.	RIWU ET AL.	STEPHENS ET AL.	YUSRANSYAH ET AL.

**D1 (Bias due to confounders)**	Low	Low	Moderate	Serious	Moderate	Serious	Low

**D2 (Bias in selection of study participants)**	Low	Low	Moderate	Moderate	Moderate	Low	Moderate

**D3 (Bias in classification of Interventions)**	Low	Low	Low	Low	Low	Low	Low

**D4 (Bias due to deviations from intended interventions)**	Low	Low	Moderate	Moderate	Moderate	Moderate	Low

**D5 (Bias due to missing data)**	Low	Low	Low	Low	Low	Low	Low

**D6 (Bias in measurements of outcomes)**	Low	Low	Moderate	Moderate	Moderate	Moderate	Moderate

**D7 (Bias in the selection of reported result)**	Low	Low	Low	Low	Low	Low	Low

**Overall assessment**	**Low**	**Low**	**Moderate**	**Serious**	**Moderate**	**Serious**	**Moderate**


## Discussion

### Cost and cost-effectiveness of hypertension management

This systematic review on cost-effectiveness and strategies for hypertension management using NPHCWs shows that cost and cost-effectiveness vary across country groups, study design, and perspectives. The implementation strategies to support the task-shifting included training and educating healthcare workers on how to screen, diagnose, manage patients with hypertension, and how to follow them up and refer those with severe cases. Strategies to support patient care by NPHCWs include community engagement, BP screening, and counselling on medication adherence, reminder SMS and lifestyle modifications. NPHCWs that provided care from the commonest to the least in the analyzed studies were pharmacists (37,38,40), followed by community health workers and nurses (35,36,39,40), and village health workers (41). The costing of interventions varied across countries and years of intervention. However, for uniformity, the costing was adjusted for inflation to 2022 equivalent of each currency using an online global rates inflation calculator before conversion to the International dollars of 2022 using the World Bank Group Purchasing Power Parity conversion factor (29,42). Only one study reported on the costs of 1 mmHg reduction in BP and hypertension control per person, while six studies reported on the annual cost of treatment. The finding is similar to the review of economic evaluations of BP monitoring techniques in patients with hypertension (43) and cost-effectiveness of hypertension management in LMICs (8). The reviews included costs of 1 mmHg reduction in SBP and or DBP, costs of controlling hypertension per patient, annual cost of managing hypertension per patient and utility costs such as DALY averted and QALY gained.

Five out of seven studies reported interventions that were very cost-effective, while the multinational study involving three countries showed cost-effectiveness within each participating country. Although two out of three studies involving pharmacists had counselling as a very cost-effective intervention, the details of the counselling were not given. Previous reviews by A-Aqeel et al., and Ahumada-Canale et al., show that pharmacist counselling were common and cost-effective intervention for medication adherence, preventing cardiovascular risks and other chronic diseases management (44,45); however, unlike Aqeel and Ahumada-Canale, our findings should be interpreted with caution in view of the limitations of the details of the counselling. There was a significant difference in the cost of QALY between patients’ and payers’ perspectives in the same study by Yusransyah et al (38). Although the reason for this difference is not clear, it is possible that the payer may have included other costs that were excluded by the patient. To mitigate against such disparities, a societal perspective is often recommended for a balanced view (46,47,48). The only study that was not cost-effective had an ICER above the Ghanaian willingness to pay threshold of $645 per DALY averted in 2017 and was also greater than three times the country’s GDP per capita in 2022. The lack of cost-effectiveness may be attributed to high overhead costs and increased frequency of visits to healthcare providers that was modeled in the intervention group. Since it was modeled and not an actual study, it may be subject to confounders and selection bias and may not be a reflection of routine practice. Like our findings, other studies have proved to be cost-effective based on their ICER and hypertension control using similar strategies (49,50,51,52,53,54).

### Implementation strategies of task shifting for hypertension management

Different implementation strategies were used to achieve the desired effectiveness and implementation outcomes of task-shifted care. Most studies use more than one strategy, allowing a focus on community members or healthcare workers. The strategies to support task-shifting included training of healthcare workers on screening, diagnosis, healthy lifestyle counselling, treatments, follow-up, and referral where necessary. Others include financial incentives to healthcare providers. Strategies used by NPHCWs to support patient care or control BP include community engagement, counseling on prevention of cardiovascular risk factors, lifestyle modifications, and sending reminder SMS for medication refill. Accurate BP measurements, lifestyle modifications, medication adherence, and financial incentives have similarly been recommended as effective strategies for hypertension management by Abdalla et al. in the United States (12). Unlike our study, the study by Abdalla et al. was conducted among physician healthcare workers. Similar to our findings, studies in LMICs, including Uganda, India, Pakistan, and Nigeria, have shown that training of NPHCWs on BP measurement and hypertension management using simplified treatment protocols, health education of stakeholders and community engagement are successful strategies in hypertension management (55,56,57,58,59,60,61,62). Other successful strategies documented in the literature but not identified in our review included telemonitoring of home blood pressure combined with pharmacist-led hypertension management (63), as well as pharmacist–physician joint interventions focusing on medication education, medication adherence, and healthy lifestyle modification (64). These may be due to high costs of implementing these strategies in LMICs.

### Policy implications

These findings have several potential policy implications in LMICs. Although the review shows that there are limited studies in this field, our findings support the cost-effectiveness of task shifting to NPHCWs, particularly the community health workers, nurses, and pharmacists mostly under the supervision of physicians to improve access to affordable and quality healthcare in resource-limited settings. Government and healthcare organizations can invest in training, certifying, and equipping NPHCWs with the required knowledge and skills to successfully prevent, diagnose, and manage hypertension. Policymakers and funding agencies should encourage future researchers to adopt standardized methods or protocols for reporting studies on cost-effectiveness to ease comparison and generalization.

### Strengths and limitations

This review has several strengths. It was a thorough evaluation of the published literature on costs and cost-effectiveness of task shifting across different LMICs and implementation strategies, and included articles published from inception to 2024 involving pharmacological and non-pharmacological interventions, giving a holistic view of hypertension management approaches. The review also provided a comprehensive assessment of available cost metrics in hypertension management, such as costs per mmHg reduction in blood pressure, cost of BP control, annual cost of treatment, cost per DALY averted, and cost per QALY gained. It also highlights the general cost-effectiveness of task shifting to NPHCWs for hypertension management in LMICs. The review also attempted to standardize costs by converting to 2022 International dollars, facilitating easier comparison across studies.

Despite these strengths, the study also has some limitations, which includes lack of consistency in the reporting of costs and cost-effectiveness across studies, making it difficult for direct comparisons. The review was limited to articles published in the English language. Some were modeled studies and not actually conducted, hence limiting the applicability of their findings to real-world settings. Some studies reported incomplete data, making it difficult to give an accurate cost-effective evaluation. There was a large degree of heterogeneity and thus only a narrative synthesis was able to be performed.

## Conclusion

In conclusion, this review showed that management of hypertension by NPHCWs, particularly community health workers, nurses, and pharmacists in some LMICs, appears cost-effective, which supports policies that promote task shifting and capacity building on NPHCWs for accessible and affordable healthcare services. However, there is a need for more studies in this area to assess cost-effectiveness in a larger number of settings for government buy-in to support scale-up and sustainability.

## Registration & Protocol

The systematic review protocol was developed in May 2024 and registered on PROSPERO (CRD42024544755).

## Data Accessibility Statement

Data is publicly available, and the full text reviewed or analyzed is listed in Supplemental Table 4.

## Additional File

The additional file for this article can be found as follows:

10.5334/gh.1533.s1 Supplementary File. Supplemental Tables 1 to 4.
