# Supplementary material for: Cost-Effectiveness and Implementation Strategies for Hypertension Management Using Non-Physician Healthcare Workers in Low- and Middle-Income Countries: A Systematic Review
Source: Glob Heart. 2026 Mar 12;21(1):18. doi: 10.5334/gh.1533 (PMC12985814; doi:10.5334/gh.1533)
Supplement: Supplementary File. — Supplemental Tables 1 to 4. [file gh-21-1-1533-s1.pdf]

Supplemental Table 1: Inclusion and Exclusion Criteria.

Metrics	Inclusion criteria	Exclusion criteria
Population	Adult 18 years & above with hypertension.	
Intervention	Interventions related to hypertension screening, diagnosis & management.<br><br>Management could be non-pharmacological such as lifestyle modifications (e.g., weight loss, exercise, sodium reduction, alcohol reduction, cessation of cigarette smoking, increased intake of fruits & vegetables) and pharmacological where blood pressure lowering medications are used.<br><br>Care offered by non-physician healthcare workers such as community health workers, village health workers, pharmacists, nurses, dieticians etc.	Hypertension in children or pregnancy.<br><br>Treatment by surgical intervention.<br><br>Hypertension secondary to other medical conditions like diabetes mellitus and chronic kidney diseases.<br><br>Interventions that included physicians
Comparator	Comparative and non-comparative studies. Comparator can be status quo (usual care) or doing nothing	
Outcomes	Cost, & or benefits of managing hypertension, implementation strategies used in the studies	
Time frame	Studies from inception to May 2024	
Settings	Studies done in low-middle income countries based on World Bank Classifications	
Study design	Studies on costs, cost effectiveness, cost benefits of managing hypertension using non-physician healthcare workers<br><br>Full articles written in English language that used primary data or modelling	Abstract, study protocols, editorials, opinion letters, case-reports, case series, correspondences & reviews

***Supplemental Table 2: Definition of relevant terms*.**

**Cost effectiveness**	Cost-effectiveness in the management of hypertension refers to the evaluation of the economic efficiency of interventions used in the prevention, detection, and treatment of hypertension, relative to their expected health outcomes. It compares the costs and health benefits of different strategies to determine which provides the best value for money spent(67, 68)
**Incremental Cost Effectiveness Ratio (ICER)**	The additional cost of one unit of outcome gained by one intervention or strategy compared with another. It is expressed as the ratio of the difference between two interventions to the difference in effectiveness(69).
**Disability-Adjusted Life Year (DALY)**	DALY is a measure that assesses the overall burden of a disease. One DALY represents the loss of equivalent of one year of full health. It is a summation of the years of life lost (YLL) due to premature mortality and the years lived with disability (YLD) due to the condition(70).
**Quality-Adjusted Life Years (QALY)**	QALY is a measure that assesses the impact of an intervention on a person's length of life and the impact on their health-related quality of life. It is a product of the number of years an individual is expected to live and a utility score (the utility score ranges from 0 which is death to one which signifies perfect health)(71).
**Gross Domestic Product (GDP)**	GDP is the comprehensive measure of the monetary value of goods and services produced in a country within a given time frame. It is the summation of the total amount added by all domestic producers in the economy plus any product taxes minus any subsidies that were not captured in the value of the products(42, 72).
**Implementation strategies**	Implementation strategies in the management of hypertension refer to the systematic methods and approaches used in integrating and sustaining evidence-based interventions into routine healthcare practice for the management of hypertension. It is aimed at improving the accessibility, effectiveness, and sustainability of the hypertension management programs in real-world settings(12-14).

**Implementation Outcomes**	Implementation outcomes are defined as the effects of intentional and calculated actions taken to implement new treatments, practices and services. It functions as indicators for implementation success and process, and as an intermediate outcomes in relation to service or clinical outcomes in effectiveness of treatment and quality of care research(26).
1. Penetration	Penetration or reach is the measure of how widely or deeply an intervention is integrated into the healthcare service system(26).
2. Fidelity	Fidelity is a measure of how closely an intervention is implemented by following the original plan or study protocol as designed(26).
3. Costs	Cost is a measure of the financial implication of what will require to implement and sustain an intervention(26, 73).
4. Adoption	Adoption is the initial decision of an individual or a healthcare provider to actually use, start using or try an intervention(26).
5. Feasibility	Feasibility is a measure of how practicable or doable is the new intervention within the healthcare settings(26).
6. Acceptability	Acceptability is a measure of the perception among stakeholders that a given intervention is agreeable or satisfactory(26).
7. Sustainability	Sustainability measures the extent to which a newly implemented intervention is maintained or institutionalized over time as part of the modus operando of the system or healthcare setting(26).
**Service Outcomes**	Is the effects of the program intervention on the quality, access, and efficiency of the healthcare service delivery. It includes efficiency, safety, effectiveness, equity, patient-centeredness and timeliness(26, 74).

Supplemental Table 3: The Medical Subject Heading (MeSH) Search Terms Used for Searching.

Hypertension	1. exp Hypertension/ or exp blood pressure/ or exp pre-eclampsia/<br>OR<br>2. (hypertens* or high-blood-pressure* or pre-eclampsia or eclampsia).ti,ab,hw,kf.
Cost-effectiveness	3. exp Cost-Effectiveness Analysis/ or exp Cost-Benefit Analysis/<br>OR<br>4. (Economic* or cost* or Return-on-investment* or budget).ti,ab,hw,kf.
Non-physician healthcare workers	5. exp Allied Health Personnel/ or exp Physician Assistants/ or exp Nurse Practitioners/ or exp Physical Therapists/ or exp Occupational Therapists/ or exp Community Health Workers/<br>OR<br>6. (community-health-worker* or allied-health-personnel* or APN or physician-assistant* or nurse-practitioner* or physical-therapist* or occupational-therapist* or non-physician-healthcare-worker* or allied-healthcare-worker*).ti,ab,hw,kf.
Low- and middle-income countries	7. (afghan* or africa* or albania* or algeria* or angola* or antigua* or barbuda* or argentin* or armenia* or aruba* or azerbaijan* or bahrain* or bangladesh* or bengal* or bangal* or barbados* or barbadian* or bajan or bajans or belarus* or belorus* or byelarus* or byelorus* or belize* or benin* or dahomey or bhutan* or bolivia* or bosnia* or herzegovin* or botswan* or batswan* or bechuanaland* or brazil* or brasil* or bulgaria* or burkina* or burkinese* or upper volta* or burundi* or urundi* or cabo verde* or cape verde* or cambodia* or kampuchea* or khmer* or cameroon* or cameroun* or ubangi shari* or chad* or chile* or china* or chinese or colombia* or comoro* or comore* or comorian* or mayotte* or congo* or zaire* or costa rica* or "cote d'ivoir*" or "cote d' ivoir*" or cote divoir* or cote d ivoir* or ivory coast* or ivorian* or croatia* or cuba or cuban or cubans or "cuba's" or cyprus* or cypriot* or czech* or djibouti* or french somaliland* or dominica* or ecuador* or egypt* or united arab republic* or el salvador* or salvadoran* or guinea* or equatoguinea* or eritrea* or estonia* or eswatini* or swaziland* or

	swazi* or swati* or ethiopia* or fiji* or gabon* or gabonese* or gabonaise* or gambia* or ((georgia or georgian or georgians) not (atlanta or california or florida)) or ghana* or gibraltar* or greece* or greek* or grecian* or grenada* or grenadian* or guam* or guatemala* or guyana* or guiana* or guyanese* or haiti* or hispaniola* or hondura* or hungary* or hungarian* or india* or indonesia* or iran* or iraq* or isle of man* or jamaica* or jordan* or kazakh* or kenya* or karabati* or korea* or kosovo* or kosova* or kyrgyz* or kirgiz* or kirghiz* or laos or lao or laotian* or latvia* or lebanon* or lebanese* or lesotho* or lesothan* or lesothonian* or basutoland* or mosotho* or basotho* or liberia* or libya* or jamahiriya* or lithuania* or macedonia* or madagasca* or malagasy* or malawi* or nyasaland* or malaysia* or malay* federation or maldives* or maldivian* or indian ocean or mali or malian* or "mali's" or malta or maltese* or "malta's" or micronesia* or marshallese* or kiribati* or marshall island* or nauru or nauran or nauruans or "naurian's" or mariana or marianas or palau or paluan* or tuvalu* or mauritania* or mauritan* or mauritius* or mexico* or mexican* or moldova* or moldovia* or mongol* or montenegr* or morocco* or moroccan* or ifni or mozambique* or mozambican* or myanmar* or burma* or burmese or namibia* or nepal* or new caledonia* or netherlands antill* or nicaragua* or niger* or oman or omani or omanis or "oman's" or pakistan* or palestin* or gaza* or west bank* or panama* or paraguay* or peru or peruvian* or "peru's" or philippine* or philipine* or phillipine* or phillippine* or filipino* or filipina* or poland* or polish or pole or poles or portugal* or portuguese or puerto ric* or romania* or russia* or ussr* or soviet* or rwanda* or rwandese or ruanda* or ruandese or samoa* or navigator island* or pacific island* or polynesia* or "sao tome and principe*" or sao tomean* or santomean* or saudi arabia* or saudi or saudis or senegal* or serbia* or seychell* or sierra leone* or slovak* or sloven* or melanesia* or solomon island* or norfolk island* or somali* or sri lanka* or ceylon* or "saint kitts and nevis*" or "st kitts and nevis*" or kittian* or nevisian* or saint lucia* or st lucia* or saint vincent* or st vincent* or vincentian* or grenadine* or sudan* or surinam* or syria* or tajik* or tadjik* or tadzhik* or tanzania* or tanganyika* or thai* or timor leste* or east timor* or timorese* or togo or togoles* or "togo's" or tonga* or trinidad* or tobago* or tunisia* or turkiy* or turkey* or turk or turks or turkish or turkmen* or uganda*

	or ukrain* or uruguay* or uzbek* or vanuatu* or new hebrides* or venezuela* or vietnam* or viet nam* or yemen* or yugoslav* or zambia* or zimbabwe* or rhodesia* or arab* countr* or middle east* or global south or sahara* or subsahara* or magreb* or maghrib* or west indies* or caribbean* or central america* or latin america* or south america* or central asia* or north asia* or northern asia* or southeastern asia* or south eastern asia* or southeast asia* or south east asia* or west asia* or western asia* or east europe* or eastern europe* or developing countr* or developing nation* or developing population* or developing world or less developed countr* or less developed nation* or less developed world or lesser developed countr* or lesser developed nation* or lesser developed world or under developed countr* or under developed nation* or under developed world or underdeveloped countr* or underdeveloped nation* or underdeveloped world or middle income countr* or middle income nation* or middle income population* or low income countr* or low income nation* or low income population* or lower income countr* or lower income nation* or lower income population* or underserved countr* or underserved nation* or underserved population* or under served population* or under served nation* or under served population* or deprived countr* or deprived population* or high burden countr* or high burden nation* or countdown countr* or countdown nation* or poor countr* or poor nation* or poor population* or poor world or poorer countr* or poorer nation* or poorer population* or poorer world or developing econom* or less developed econom* or underdeveloped econom* or under developed econom* or middle income econom* or low income econom* or lower income econom* or low gdp or low gnp or low gross domestic or low gross national or lower gdp or lower gnp or lower gross domestic or lower gross national or lmic or lmics or third world or lami countr* or transitional countr* or emerging econom* or emerging nation*).ti,ab,hw,kf.

Supplemental Table 4. References of Included Studies.

S/No	Study
**1**	Finkelstein EA, Krishnan A, Naheed A, Jehan I, de Silva HA, Gandhi M, Lim CW, Chakma N, Ediriweera DS, Khan J, Kasturiratne A, Hirani S, Solayman AKM, Jafar TH; COBRA-BPS study group. Budget impact and cost-effectiveness analyses of the COBRA-BPS multicomponent hypertension management programme in rural communities in Bangladesh, Pakistan, and Sri Lanka. Lancet Glob Health. 2021 May;9(5):e660-e667. doi: 10.1016/S2214-109X(21)00033-4. Epub 2021 Mar 19. PMID: 33751956; PMCID: PMC8050199.
**2**	Garcia-Pena, C; Thorogood, M; Wonderling, D; Reyes-Frausto, S; (2002) Economic analysis of a pragmatic randomised trial of home visits by a nurse to elderly people with hypertension in Mexico. Salud publica de Mexico, 44 (1). pp. 14-20. ISSN 0036-3634 DOI: https://doi.org/10.1590/S0036 36342002000100002
**3**	Gaziano, T.A., Bertram, M., Tollman, S.M. *et al.* Hypertension education and adherence in South Africa: a cost-effectiveness analysis of community health workers. *BMC Public Health* **14**, 240 (2014). https://doi.org/10.1186/1471-2458-14-240
**4**	Pozo-Martin F, Akazili J, Der R, Laar A, Adler AJ, Lamptey P, et al. Cost-effectiveness of a Community-based Hypertension Improvement Project (ComHIP) in Ghana: results from a modelling study. BMJ Open. 2021 Sep 2;11(9):e039594. doi: 10.1136/bmjopen-2020-039594. Erratum in: BMJ Open. 2021 Oct 5;11(10):e039594corr1. doi: 10.1136/bmjopen-2020-039594corr1. PMID: 34475137; PMCID: PMC8413878.
**5**	Riwu M, Yubiliana G, Halimah E, Diantini A. Cost-Effectiveness Analysis of Pharmacist Counselling in Therapeutic Success and Quality of Life of Hypertensive Patients. Research J. Pharm*. and Tech. 2019; 12(12): 5845-5847. doi: 10.5958/0974-360X.2019.01013.8*
**6**	Stephens JH, Addepalli A, Chaudhuri S, Niyonzima A, Musominali S, Uwamungu JC, et al. (2021) Chronic Disease in the Community (CDCom) Program: Hypertension and non-communicable disease care by village health workers in rural Uganda. PLoS ONE 16(2): e0247464. https://doi.org/10.1371/journal. pone.0247464
**7**	Yusransyah, Halimah, E. ., & Suwantika, A. A. . (2022). IAI SPECIAL EDITION: Optimal scenario of antihypertension's cost-effectiveness in Prolanis hypertension patients: A case study of Pandeglang District, Indonesia. *Pharmacy Education*, *22*(2), p. 85–91. https://doi.org/10.46542/pe.2022.222.8591
